# A unified component-based data-driven framework to support interoperability in the healthcare systems

**DOI:** 10.1016/j.heliyon.2024.e35036

**Published:** 2024-07-23

**Authors:** Amir Torab-Miandoab, Taha Samad-Soltani, Ahmadreza Jodati, Fariborz Akbarzadeh, Peyman Rezaei-Hachesu

**Affiliations:** a Department of Health Information Technology, School of Management and Medical Informatics, Tabriz University of Medical Sciences, Tabriz, Iran; b Cardiovascular Research Center, Tabriz University of Medical Sciences, Tabriz, Iran

**Keywords:** Component-based, Data-driven, Framework, Interoperability, Healthcare systems

## Abstract

Healthcare organizations must urgently prioritize interoperability to enhance the quality of care they provide. However, achieving this collaboration comes with numerous challenges, including differing approaches, data formats, and standards, as well as concerns about privacy, security, technical complexity, and legal and regulatory issues. To tackle these challenges, we determined a set of interoperability solutions. We also developed a comprehensive, component-based, data-driven framework for healthcare systems. Our study's approach involved three main steps: first, conducting a literature review to gather interoperability requirements and solutions from online databases and grey literature; second, carrying out a qualitative study to develop a framework based on the review results and focus group discussions; and third, using the Delphi method to validate the framework with experts. We extracted information from 36 articles during the screening and assessment process. Based on the proposed framework, we organized the identified themes into various categories, including architecture, architecture components, standards, platforms, policies, data sources, consumers, applications, level of interoperability, healthcare facilities, and considerations. Experts believe that establishing a comprehensive architecture for launching interoperability between health information systems can greatly facilitate this process. All framework components (totaling 197) received unanimous approval. The landscape of healthcare delivery is shifting from a focus on diseases to a patient-centered, data-driven approach. There is a growing demand for personalized healthcare systems, which necessitates increased interoperability among all healthcare stakeholders, particularly when dealing with diverse types of data. Our framework is designed to facilitate the implementation of various types of interoperability in healthcare systems.

## 1 Introduction

The capacity of different software, hardware, or systems to exchange information and communicate with each other without any difficulties or compatibility problems is known as interoperability [1]. There are different levels of interoperability, including syntactic, technical, semantic, organizational, and legal [1]. The capacity of various systems to communicate and share data with one another through common protocols, data formats, and interfaces is referred to as technical interoperability [1]. The ability of various systems to comprehend and interpret the structure and format of data shared amongst them is known as syntactic interoperability, and it can be attained by using standardized data formats and syntax [1]. The ability of various systems to comprehend the context and meaning of data shared amongst them through the use of common ontologies, taxonomies, and vocabularies is known as semantic interoperability [1]. The ability of several organizations or entities to collaborate successfully and economically by utilizing shared policies, procedures, and processes is known as organizational interoperability [1]. The capacity of various systems to abide by legal and regulatory requirements when exchanging data is known as legal interoperability, and it can be accomplished by using common standards and rules [1].

In today's interconnected world, interoperability holds great significance across various sectors, including finance, energy, transportation, technology, telecommunications, and healthcare. It facilitates the seamless sharing of data and efficient collaboration among different systems [2]. In the finance industry, interoperability plays an important role in enabling secure and efficient transactions between various financial institutions, payment systems, and currencies. It empowers customers to seamlessly transfer funds between accounts, make payments, and conduct other financial transactions [3].

Within the energy industry, interoperability is critical for effectively managing and optimizing the distribution and consumption of energy across diverse systems, such as power grids, renewable energy sources, and smart homes. It empowers energy providers to monitor and control energy usage, reduce waste, and enhance overall efficiency [4]. In the transportation sector, interoperability is essential for ensuring the safe and efficient movement of people and goods across different modes of transportation, including air, land, and sea. It enables diverse transportation systems to communicate and exchange crucial data, such as traffic information, weather conditions, and cargo tracking [5].

In the technology industry, interoperability is crucial for ensuring the effective collaboration of different software and hardware systems. For instance, in the realm of cloud computing, interoperability allows different cloud platforms to communicate, enabling users to seamlessly move data and applications between various cloud providers [6]. Within the realm of telecommunications, interoperability is vital for enabling different devices and networks to communicate with each other. This becomes increasingly important as the quantity of linked gadgets keeps increasing, necessitating seamless collaboration among different devices and networks to provide a smooth user experience [7].

Health information systems are used in healthcare settings all over the world, and in the last few decades, a wide range of technologies have been developed to convert paper-based health information into electronic health information [8]. Healthcare organizations can gather, store, manage, analyze, and optimize patient treatment histories and other crucial data with the use of a health information system [9]. These tools also make it easier for healthcare professionals to obtain data on macro environmental issues, like community health trends, and they enhance the efficacy and efficiency of health services by facilitating improved administration at all health service levels [10].

To achieve these benefits, the ability to support decision-making based on evidence retrieved from big, heterogeneous, or distributed systems is critical [11]. On the one hand, these systems are typically private, can differ between medical facilities, and are designed for local access; yet, on the other hand, patient health data might be shared among an arbitrary number of medical facilities [12,13]. Interoperability across systems can thus be defined as their ability to exchange semantically consistent information and data that can be used by all [14]. Financial resources may be wasted and patient treatment may be of worse quality if these systems are not interoperable. In order to facilitate widespread access to patient health information and better health care coordination, it is imperative that integration methods be established across the various health information systems [15,16].

Interoperability is particularly important in the healthcare domain because it involves the exchange of critical and sensitive patient health information between different healthcare providers, systems, and devices. Additionally, the healthcare industry has unique challenges when it comes to interoperability, such as variety of systems, different data formats and types, huge amounts of data, privacy and security concerns, technical complexity, and legal and regulatory challenges. While there may be some similarities in the technical aspects of interoperability across different domains and industries, the specific challenges and requirements can vary significantly depending on the context [17].

Various approaches to achieving interoperability in health information systems have been suggested [13]. However, the implementation of interoperability necessitates conducting studies in this field to leverage the successful experiences of countries. This will help determine which solutions should be prioritized or disregarded. It is crucial to emphasize that government representatives, legislators, software developers, informatics specialists, and health IT professionals will gain knowledge from the presentation of the conceptual framework for interoperability in healthcare systems about how to apply and plan solutions for implementing interoperability in healthcare systems [18].

Consequently, this study presents a unified component-based data-driven framework to support interoperability in healthcare systems. In this work, we first provide an outline of the state of interoperability in healthcare and other industries. We then depict the methodology of our study to reach the framework. Finally, we describe the components of our proposed framework and how they work together to support interoperability in healthcare settings. Through this paper, we aim to provide a comprehensive and practical solution to support interoperability in healthcare systems, ultimately improving the quality of care for patients.

## 2 Materials and methods

**Fig. 1:**
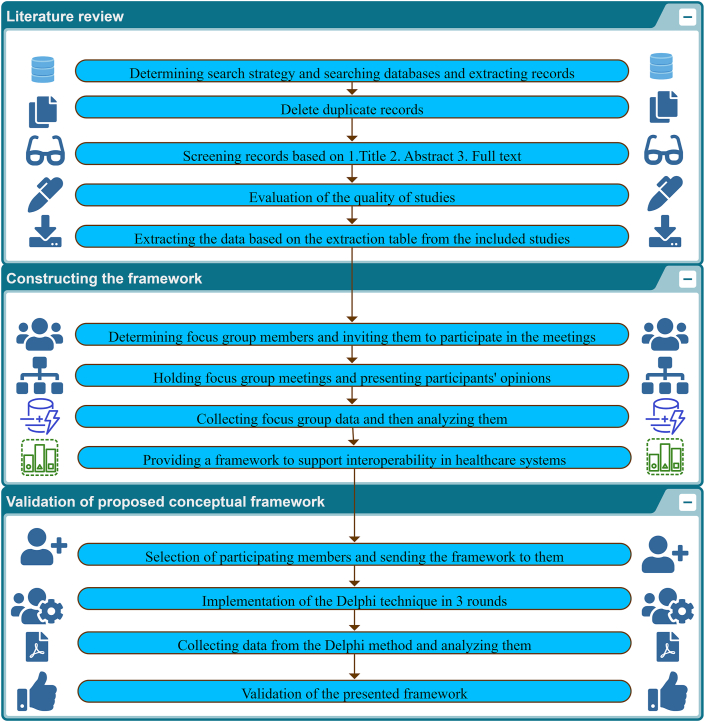
An overview of the methods steps.

The current investigation's technique comprises three phases (see Fig. 1). Four authors screened the papers from the information sources using inclusion and exclusion criteria. They read the full text of the articles presenting various approaches for implementing interoperability in the healthcare environment. Information from 36 articles was extracted. The results of the focus group and the subsequent phase were utilized to develop an initial framework, which was then forwarded to specialists for completeness and confirmation.

### 2.1 Phase 1. Literature review

Preferred Reporting Items for Systematic reviews and Meta-Analyses (PRISMA) was followed in conducting the literature study in order to find papers that detailed the application of interoperability in healthcare ecosystems. Based on relevant papers and suggestions from professionals in medical informatics, health information technology, and health information management, we chose relevant keywords. The search approach was approved by a knowledgeable librarian. The resulting syntax is as follows:

(“Electronic Medical Record" OR “EMR” OR "Electronic Health Record" OR “EHR" OR "Computerized Medical Record" OR “CMR” OR "Automated Medical Record" OR “AMR” OR "Hospital Information System" OR "Health Information System" OR “HIS” OR "Clinical Information System" OR “CIS” OR "Medical Record System") AND Interoperability

Studies were included up to October 25, 2022. The selection process involved searching official Iranian websites (such as the Ministry of Health and Medical Education) as well as online databases PubMed, Web of Science, Scopus, IEEE, ProQuest, MEDLINE, Cochrane Library, Embase, Scientific Information Database (SID), IRAN MEDEX, Islamic World Science Citation Center (ISC), and Google Scholar. To prevent publishing bias and to make sure that the most articles were included, online books, published articles, conference abstracts, seminar materials, and reference books were all included in the searches. Additionally, manual searches for potentially relevant research took place in the references and bibliographies of published articles and reviews. An email alert mechanism in the electronic databases was developed to keep track of any recently published papers that satisfied the selection criteria based on the search history retained until October 25, 2022.

Articles that addressed health information system development, deployment, validation, or translation in the framework of interoperability were deemed acceptable. Review articles, peer-reviewed research, and English-language papers were all included in this analysis, and full text access was made available for all of them. Opinions, protocols, and studies irrelevant to the subject were excluded, as well as letters to the editor. The number of publishing years was unlimited.

The identified publications were uploaded to an EndNote X8 collection, and duplicate publications were eliminated. After removing the duplicates, the articles were given to four authors (A TM, T SS, A J and P RH) separately and independently using a spreadsheet, and the opinions of the authors were first independently obtained based on the title and inclusion and exclusion criteria and articles that received 3 or more votes for deletion were selected and deleted. In the next step, the remaining articles were reviewed independently by the authors based on the abstract and inclusion and exclusion criteria and the candidate articles for removal were selected and removed based on 3 or more votes of the authors. Finally, the opinions of the authors on the remaining articles were independently obtained based on the full text and inclusion and exclusion criteria and the candidate articles for removal were selected and removed based on 3 or more votes of the authors. Disagreements were resolved by the fifth author (F A) through an online meeting. Authors, interoperable systems, architectures and components, processes, standards, platforms, settings, levels of interoperability, information resources, and the extent of implementation were all extracted from the data.

The quality of the included research was assessed in order to bolster the inclusion/exclusion process. The used critical appraisal checklist was created by combining the Quality Appraisal of Reliability Studies (QAREL) and the Quality Assessment of Diagnostic Accuracy Studies (QUADAS) tools. This instrument was designed to assess studies that combined validity and reliability testing, or to assess validity and reliability testing independently [19,20]. There are 13 items on the list. Four researchers conducted a risk of bias analysis on each of the listed papers. Research papers with a 60 % or above were considered to be of high quality.

### 2.2 Phase 2. Constructing the framework

The identified individuals were found using the purposive sampling technique. Through the prior literature search on interoperability and health information systems in databases, potential participants were found. Participants in this study had to be specialists in health information management, health information technology, medical informatics, information technology, and providing health care services. They also needed to be Iranian citizens, have at least three articles published in the last ten years on health information systems and interoperability, and have worked in a related field for at least five years. Eligible participants were invited to participate in focus groups via social media. Focus group sessions were conducted between November 2022 and December 2022.

A total of 12 participants took part across three focus groups. Each group was formed of 10–12 participants. Focus group sessions took place at the health information technology laboratory, faculty of management and medical informatics, Tabriz University of Medical Sciences. Participants received an information statement about the study. They were offered time to join the focus group. Before the focus groups sessions were conducted, all participants provided written consent to participate. Participants could freely leave the study at any stage. All focus group sessions were facilitated in person by one researcher (A TM). Focus group sessions were audio-recorded. Each focus group session lasted for approximately one-and-a-half hours.

The focus group meetings included open-ended questions along with a series of probing questions in addition to the extracted results from the first phase. Although they responded to questions separately, participants were urged to interact with one another. The digital recordings of the focus groups' sessions were verbatim transcribed after being deidentified. The facilitator checked the accuracy of each transcript. Three steps of an inductive thematic analysis were used to examine the transcribed text. In order to become more familiar with the material and build an impression of the overall content, researchers first listened to audio recordings before reading the text out loud many times. Second, by locating terms and phrases in the transcripts, a preliminary coding system was created. Third, codes were assembled into potential themes that underwent ongoing assessment and improvement. To support the findings, codes and themes were created in constant dialogue with the study team. Every focus group transcript was coded by one researcher (A TM), and another researcher (T SS) also coded focus group transcripts. Interviews that were double-coded were compared, and disagreements were settled by consensus. Using MAXQDA (version 22), a qualitative data analysis program, coding and analysis were aided. Finally, a unified component-based data-driven framework for support interoperability in healthcare systems was created.

### 2.3 Phase 3. Validation of proposed conceptual framework

The Delphi methodology was used to validate the basic framework. Delphi studies are a well-known research technique that uses questionnaires with feedback in between to help a panel of expert's reach consensus on a certain topic across several rounds. According to the Delphi method, experts should be anonymous during the research to prevent bias from conflict or the need to defend preconceived ideas. The research team anonymizes, compiles, and shares the expert opinions and input with the panel for evaluation until the predetermined termination criteria are satisfied in each round. In Delphi surveys, consensus is frequently defined in terms of percentage agreement. An acceptable degree of consensus has been determined to be 70 % agreement. Over the years, other Delphi study configurations have been proposed, including classical, policy, decision, and ranking-type Delphi. The associated rounds may be centered around rating, narrowing down, brainstorming, or validation. Several rigor criteria and best practices have been suggested to guarantee the appropriate application of the Delphi technique, which we witnessed during the course of our Delphi study. These requirements consist of, among other things, giving clear directions at every stage of the study, guaranteeing anonymity throughout, and disclosing pertinent background data on the experts' backgrounds and demographics [21].

Experts were contacted using a purposive sampling (non-probability) strategy. Participants were drawn from six distinct groups, including health information management, health information technology, information technology, physicians, nurses, and medical informatics, to assure coverage across specialist domains. Potential experts were identified through the previous literature search on interoperability and health information systems in databases. Experts who lived in Iran, had at least three publications published in the last ten years on health information systems and interoperability, and had five years or more of professional experience in a related subject were eligible to participate in this study. In order to achieve the best combination of decision quality and data manageability, we aimed for 30 respondents. By email, participants were notified. Despite maintaining their anonymity to one another, participants were not anonymous to the researcher. Participants received an information statement about the study and they could freely leave the study at any stage. Each subject gave their written consent. Delphi participants were different from those who participated in the focus group.

During the first phase of the Delphi process, the experts were given the first framework in the form of a questionnaire that contained all of the framework's components. Consensus was sought about the conceptual framework given during the first Delphi round. The responses were rated on a 5-point Likert-type scale, with 1 being the least relevant and 5 being the most relevant, with the option for additional remarks. The opinions of the experts were analyzed using thematic analysis. The proposed framework was modified. After attaining agreement, the second round's main goal was to assess how well and concisely the framework had been put out. In the second stage, the modified framework was again presented to the experts in the form of a questionnaire. A 5-point Likert scale was used to score these items, with 1 representing “strongly disagree” and 5 representing “strongly agree.” In the third phase of the Delphi process, the experts were shown the revised framework. The third round concentrated on areas where the previous rounds' participants had not yet reached agreement. Also, participants were asked to assess the appropriateness of inclusion. These elements were modified in response to professional opinion.

Data was collected in January 2023. Excel was used to perform all of the analyses. For all questionnaire items, the acceptable values for the framework to be accepted are the mean over 3.5 and the standard deviation less than 1. Measures of central tendency and dispersion, such as mean, median, and standard deviation, were employed to examine the data. During each Delphi stage, participants who had not (fully) completed the survey received two reminders in an effort to enhance response rates.

## 3 Results

### 3.1 Phase 1. Literature review

**Fig. 2:**
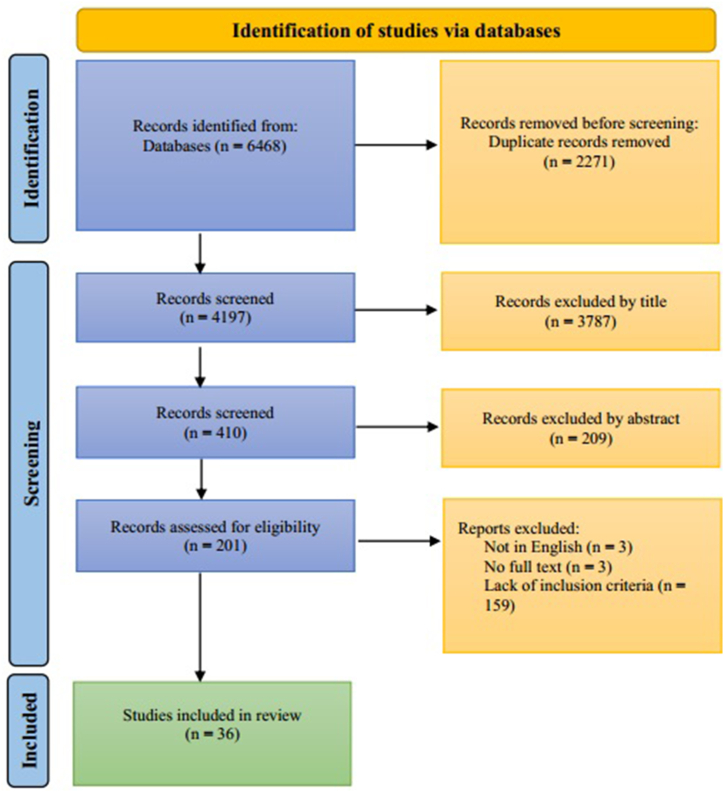
Flowchart for the selection process of the included studies.

4197 titles remained after duplicate articles were eliminated out of the total 6468 titles that were pulled from the databases. When four reviewers reviewed the titles and abstracts for appropriateness, they removed 3787 papers that they determined were unrelated to the review's topic. On the basis of titles, 410 articles were chosen. 209 papers were disqualified after the abstracts were examined because they failed to meet the criteria set forth. A total of 201 full-text publications were located and evaluated in accordance with the requirements. 36 articles were included in the review after 165 articles were excluded (see Fig. 2) due to the assessment.

Table 1 provides a summary of the key elements for implementing interoperability based on the findings. This can be classified into nine categories: (1) systems, (2) architectures and components, (3) processes, (4) standards, (5) platforms, (6) settings, (7) level of interoperability, (8) information resources and (9) extent of implementation. Table 1 details these classifications and their associated cases.

**Table 1:** Details of the selected studies in this review.

Authors	Interoperable systems	Architecture and components	Processes	Standards	Platforms	Level of interoperability	Extent of implementation	Settings	Information resources
Kim HS et al. [22]	EMR, PHR and insurance system	Web service 1) storage layer, 2) boundary object layer, 3) application program layer, 4) user interface layer	Data entry, searching, removing, editing, exchange, mapping and linkage between terminologies, clinical decision-making support and calculation of reimbursement charge	-Terminology: SNOMED-CT, ICD, CPT, ABC, NANDA, NOC, NIC, LOINC, HCPCS and CCC -Content: CDA -Transport: HL7 -Security: HIPAA	-Hardware: server and PC -Technology: XML and Java	Functional	National	Ambulatory care centers	Centers for Medicare and Medicaid services
Plácido GR et al. [23]	Health information systems, mobile devices, biosensors and a set of primitives such as medical devises	Service-oriented architecture 1) procedural layer, 2) documental layer	Documentation, exchange of information, medication and telemedicine	-Transport: SOAP, UDDI, WSDL, Zigbee	- Hardware: server, mobile device, biosensor, set-top box, PDA, medical devices, ADSL, cable and television - Software: Java RMI - Technology: SANDS, REST, Bluetooth, RFID, internet network, PAN and infrared	Structural	National	Hospitals, primary care centers and medical emergency centers	Portugal national health service
Ciampi M et al. [24]	EHRs	Service-oriented architecture 1) EHR services, 2) repository, 3) registry, 4) cross-border services, 5) security, 6) public connectivity system	Exchange of health information, searching, retrieving, creating or updating and invalidating	- Content: CDA - Terminology: CIDOC CRM, ICD9-CM, LOINC and ATC - Transport: HL7 FHIR, IHE ITI-18-42-43-62 and SOAP	- Technology: IHE XDS and XML	Functional	National	All health care settings	National research council of Italy and the agency for digital Italy
Chen X et al. [25]	EHRs	Archetypes-based 1)reference model, 2) archetypes	Exchange of demographic identities	- Transport: LDAP and HL7 - Content: open EHR, ISO/TS 22220:2009 - TC215, ISO/DTS 27527:2007 - TC215, CDA, CEN/TS 14796	- Technology: AOM, ADL and SAML	Structural	National	All health care settings	HL7 RIM and ISO demographics
Centobelli P et al. [26]	Health care systems and sensors	Block chain-based 1) patients, 2) technological devices, 3) healthcare providers, 4) health care setting, 5) cloud database, 6) block chain network	Record, update and share of health data	- Transport: DICOM and HL7 FHIR - Security: GDPR and HIPAA - Terminology: ICD 10, ICD 11, NDC, SNOMED-CT and LOINC	- Hardware: wearables and mobile - Software: GraphQL - Technology: block chain, REST, TCP, API, IOT and cloud computing	Structural	National	Laboratories, radiology, pharmacies and hospitals	N/A
Mantas J [27]	HISs	Web services 1) Web service of data mediation, 2) User interface, 3) Knowledge base, 4) Referential ontology, 5) Mediator	Exchange semantically data	- Transport: DICOM, HL7, CEN TC2514, IEEE 11073 and EDIFACT - Content: CEN/ISO 13606 and CDA - Terminology: SNOMED-CT, UMLS and LOINC	- Software: semantic web tools - Technology: ontology and XML	Semantic	National	Hospitals	RIM
Oliveira EC [28]	PHR and national EHR	Service-oriented architecture 1) Demographic services, 2) Health Service Bus, 3) Security services, 4) Semantic framework, 5) Clinical information repository services	Exchange, store, index, retrieve, search and access data	- Content: open EHR, IHE XSD.b and IHE PIXv3 PDQv3 - Transport: SOAP, HL7 and WSDL - Terminology: SNOMED-ICD10, tabela unificada (SICTAP, CBHPM, TUSS), ICPC and LOINC - Security: WS-security	- Software: Oracle - Technology: REST, CDR, AQL and XML	Semantic	National	Primary care centers and hospitals	Open EHR which were customized with the Brazilian EHR
Janaswamy S and Kent RD [29]	EMR or EHR systems	XML-based 1) Analyzing the attributes using standard Vocabularies, 2) Making of Hybrid data model, 3) Data mapping	Data storage, access, exchange and extraction	- Transport: HL7 - Terminology: LOINC	- Software: Java software platform, Microsoft access database, MySQL, - Technology: API and XML	Semantic	National	Hospitals	N/A
Hidayat IF and EHRmanto BR [30]	Primary health centers EHR systems	Client server 1) Server layer: EHR of primary health centers and HAPI FHIR server, 2) Client layer: otEHR healthcare systems (FHIR REST client, application code and FHIR resource handler)	Create, read, update, delete and exchange of data	- Transport: HL7 FHIR - Terminology: ICD 10, ICD 9 CM, SNOMED-CT and CPT - Content: CDA	- Technology: API, json and java and REST	Semantic and syntactic	Local	Primary health centers	FHIR resource
Miranda M et al. [31]	Between HIS	Multi-agent 1) containers include: HL7 server agent, HL7 event generate agent, HL7 client agent, HL7 event process agent, IS wrapper agent, 2) consolidation data base, 3) web service tier, 4) HL7 compatible information systems	Exchange of data	- Transport: HL7 - Content: open EHR	- Software: Java agent development framework - Technology: XML	Syntactic		Hospitals	N/A
Garde S et al. [32]	Between EHRs	Archetype-based 1) Web-based management, 2) Templates and archetypes, 3) Clinical guideline, 4) Clinical knowledge artefacts, 5) Reference model	Information sharing and decision support	- Content: open EHR, ISO 13606, CCR	N/A	Semantic	National	All health care settings	Open EHR
Marcheschi P et al. [33]	Between ECG system and EHRs	Service-oriented architecture 1) common services (registry), 2) client infrastructure (Document Source), 3) network services infrastructure	Exchanging document	- Transport: HL7, DICOM and IEEE - Content: CDA - Terminology: LOINC	- Software: Adobe PDF, Adobe SVG and PACS - Hardware: ECG, XML and Holter monitoring device, server and computer	Functional	National	All health care settings	HL7 RIM
Andersen B et al. [34]	Surgical devices and CIS	service-oriented architectures 1) device observation reporter, 2) medical device network communication, 3) clinical IT network communication, 4) CIS, 5) medical device, 6)Transformation	Data collection, transformation and reporting	- Transport: HL7, and ISO/IEEE11073 - Content: CDA	- Software: Debian GNU/Linux 7.8 operation system, Oracle VM Virtual Box 4.3.18, OpenJDK 7u75, PostgreSQL 9.1, Apache Tomcat 8.0.14, Iceweasel 31.6, Chromium 41.0 and OSC Lib 0.90b - Hardware: server - Technology: Model-view-controller pattern, Java and virtual machine	Semantic	National	Hospitals	German federal ministry of education and research
Franček P et al. [35]	EHR and PHR	Cloud-based 1) HER, 2) PHR, 3) local clinical systems, 4) adapter	Data sharing	- Content: CDA and CCD - Transport: DICOM	- Software: PACS - Hardware: server, computer, mobile - Technology: API, XML, Java and REST	Semantic	National	All healthcare settings	HL7 RMIM
Kopanitsa G and Ivanov A [36]	Laboratory systems and HIS	Web-based 1) LIS, 2) HIS, 3) interface	Data exchange	- Terminology: LOINC - Transport: HL7 FHIR and CEN/ISO EN13606 - Content: CCR and open EHR	- Technology: API and REST	Semantic	National	Laboratories and hospitals	open EHR
Pintea R, et al. [37]	Cardiology department systems and HIS	service-oriented architectures 1) hospital network, 2) database, 3) interface, 4) HIS, 5) cardiology department systems	Storing and exchanging data and metadata	- Transport: HL7, SOAP and DICOM - Content: IEEE 1420.1	- Software: XML, SQL server and Java - Hardware: server, computer, smart phone and tablet - Technology: XML and Java	Semantic	Local	Hospital	Basic interoperability data model
Amr MF [38]	Between HIS	Web service 1) data base in cloud, 2) medical sectors, 3) web service, 4) query module, 5) file conversion server, 6) email server, 7) file sharing system	Data collection, consolidation, exchange and storage	N/A	- Hardware: fax and server - Technology: cloud computing, XML and JSON	Functional	Local	Hospitals	N/A
Vargas B and Ray P [39]	Between CIS	object-oriented 1) client, 2) coding and encoding server, 3) middleware, 4) HL7 messages generator, 5) interface, 6) server	Exchange of data	- Transport: HL7, DICOM and CORBA - Content: OMG and COM	- Software: java toolkit, Delphi, java virtual machines and SQL - Technology: API, visual basic and C++	Semantic	National	Hospital	N/A
Adel E et al. [40]	Between CIS	Fuzzy ontology architecture 1) heterogeneous data source, 2) local ontologies construction, 3) global fuzzy ontology construction n (rules and terminology), 4) user application interface (query, report and DSS)	Information sharing	- Transport: HL7 - Content: CEN/ISO 13606 and open EHR - Terminology: SNOMED-CT	- Software: protégé, Excel, MySQL, OntoGraf, ArchMS, XTR-RTO, xml2owl and X2OWL - Technology: OWL, RDF, ADL and XML	Semantic	Local	All healthcare settings	Open EHR
Lete SA et al. [41]	Between EMR	Archetypes-base 1) reference model (data types, data structures, identifiers and patterns), 2) conceptual model (archetypes and templates)	Exchange of data	- Transport: HL7 FHIR - Content: CEN/ISO 13606 and open EHR - Security: ISO/TS 14441 - Terminology: SNOMED CT and ICD-10	- Software: Mongo DB, XQuery, SQL, OQL, W3C and XPaths - Technology: ADL, XML and JSON	Semantic	National	All healthcare enterprises	Open EHR
Angula N and Dlodlo N [42]	Between HIS	Web-based 1) HIS, 2) middleware, 3) registry, 4) repository, 5) dashboard	Exchange important disease-surveillance information	- Transport: HL7, IHE, DICOM, NCPDP and IETF - Terminology: LOINC - Content: ISO and CDA - Security: ASTM	- Software: SQL - Technology: XML and HTML	Semantic	National	Hospitals	HL7 massaging structure
Kasthurirathne SN et al. [43]	Between EMR	Web services 1) FHIR web layer (controller and resource), 2) FHIR API layer, 3) service layer	Data exchange	- Transport: HL7 FHIR - Content: CDA - Security: OAuth - Terminology: LOINC	- Software: Mozilla - Technology: REST, XML, JSON, HTTP and API	Semantic	National	All healthcare enterprises	Open MRS
Park KS et al. [44]	Mobile equipment and the existing hospital data system	Local network 1) device connectivity management agent, 2) connection management agent, 3) interface layer, 4) integrated gateway agent, 5) domain (equipment, data management system and hospital data system), 6) massage management module, 7) rule management module	Data exchange	- Transport: HL7, POCT1-A2 and LIS2-A, LIS2-A2	- Software: Visual Studio, MS SQL, .Net Framework 2.5, Microsoft Windows XP and Microsoft Windows Server - Hardware: server - Technology: C# language	Structural	National	Hospital	N/A
Jabbar R et al. [45]	Between HIS	Block chain-based 1) HIS- front-end layer (portals for medical facility), 2) HIS-back-end layer (web or API server, medical storage), 3) block chain layer (EtEHReum private cloud), 4) access management system	Collecting, storing and sharing data	- Terminology: ICD 10 - Transport: DICOM	- Software: SQL server, CDS and PACS - Hardware: network hardware, printers, workstations, servers and scanners - Technology: API	Semantic	Local	Hospitals	N/A
Berges I, et all [46]	Between HIS	Ontology-based 1) repository of each healthcare institution, 2) application ontologies (convert DB to ontology module), 3) canonical ontology (mapping ontology module)	Data sharing	- Content: open EHR, ISO 13606 and CDA - Transport: HL7 - Terminology: SNOMED-CT and LOINC	- Technology: OWL, XML and ADL	Semantic	Local	All healthcare enterprises	Open EHR
Martínez-Villaseñor M, et all [47]	Between PHR	Ontology-based 1) profile suppliers (source document), 2) matching module, 3) profile consumer (input requirement)	Data sharing	- Content: ISO/TR 20514, ISO 13606 and CDA - Transport: HL7, CEN/TC 251 and DICOM	- Software: Microsoft HealthVault - Technology: API, XML, JSON and RDF	Semantic	National	All healthcare enterprises	Open EHR
Beštek M and Stanimirović D [48]	Between EMR	Multi-approach 1) national E-health, 2) healthcare provider, 3) cloud gateway, 4) mobile gateway, 5) patient device	Health information exchange	- Content: open EHR - Transport: Continua, HL7 FHIR and IHE - Terminology: SNOMED-CT, ICD 10 and LOINC	- Technology: REST, XML, JSON and ADL	All level	National	All healthcare enterprises	Open EHR
Sachdeva S and Bhalla S [49]	Between EMR	Archetype-based 1) database schema, 2) clinical model, 3) user, 4) expert, 5) semantic conformance (ADl, archetype and template language), 6) reference model	Data exchange	- Content: open EHR, CDA, CEN/TC251 and ISO 13606-2 - Transport: HL7, DICOM, IEEE, IHTSDO and IHE - Terminology: SNOMED-CT, ICPM, ICD 10 and LOINC - Security: ASTM	- Technology: XML, HTML, UML, JSON and ADL - Software: SQL server and XQuery	Semantic	National	All healthcare enterprises	Open EHR
Marcos M, et all [50]	Between CDSS and EHR	Archetype-based 1) reference model (generic properties and structure of information), 2) archetypes repository (clinical concepts), 3) link EHR integration engine, 4) clinical database, 5) link EHR editor engine (mapping, archetypes and terminology), 6) link EHR transformation engine	Data exchange and decision support	- Transport: HL7 and CEN/ISO EN13606 - Content: CDA, open EHR and CCR - Terminology: SNOMED-CT	- Technology: AOM, OWL, ADL and XML - Software: SQL server and XQuery	Semantic	National	Hospitals	Reference model
Bahga A and Madisetti VK [51]	Between EMR	cloud-based 1) infrastructure services layer, 2) information services layer (data integration engine), 3) application services layer (such as terminology services etc.), 4) presentation services layer (healthcare applications)	Store and transfer and access data	- Transport: HL7, DICOM, ANSI X12 and NCPDP - Content: CDA, CCR and CCD - Security: HIPAA, HITECH and OAuth	- Software: MySQL, Mirth Connect, Java software platform, load balancers, Hadoop master and oracle - Technology: API, cloud computing, XML and REST - Hardware: server, PC, slave nodes and network equipment	Semantic	Local	All healthcare enterprises	Open EHR reference model and archetype model
Ciampi M et al. [52]	Between HIS	Service oriented architecture 1) connectivity layer (connection infrastructure), 2) component layer (infrastructural components), 3) business layer (application services)	Exchange of medical data	- Transport: HL7, DICOM and CORBA - Content: open EHR, CDA and CEN/ISO EN 13606 - Security: OASIS	- Technology: XML, SSL, Java, HTTPS and IHE XDS - Software: apache MQ	Semantic	National	All healthcare enterprises	RIM
del Carmen Legaz-García M et al. [53]	Between clinical systems and EHR	Web based 1) acquisition layer (convert EHR data to archetype-ontology), 2) repositories layer (primary data about the clinical archetypes and extracts), 3) exploitation layer (services for the exploitation of the archetypes and the EHR data)	Data exchange	- Content: CEN/ISO 13606 and open HER - Transport: HL7 FHIR - Terminology: SNOMED-CT	- Technology: semantic web, ontology, ADL, OWL, XML, RDF and apache Lucene API - Hardware: computer - Software: archetype management system, SPARQL, semantic web integration tool, ontology pre-processing language version 2, LinkEHR and Protégé	Semantic	National	Hospitals	open EHR
Khan WA et al. [54]	Between HIS	Cloud based 1) consumer applications, 2) mapping execution environment (content handler, conversion manager, pattern), 3) mapping authoring environment (mediation bridge ontology, accuracy mapping engine, repository)	Information exchange	- Content: CDA, Arden Syntax, CEN/ISO 13606 and open HER - Transport: HL7	- Technology: ontology, CDSS and UMLS - Software: LinkEHR	Semantic	Local	Hospitals	RIM
Mukhiya SK and Lamo Y [55]	Between EHR	Service oriented architecture 1) open MRS server, 2) resource server, 3) authorization server, 4) patient app, 5) provider app, 6) translator	Information exchange	- Transport: HL7 FHIR - Content: CDA and Open HER - Terminology: SNOMED-CT, RxNorm, ICD 10 and LOINC - Security: OAuth	- Software: GraphQL, JavaScript, MySQL, Apache Tomcat, Hibernate and SQL server - Technology: REST, API, XML, JSON and HTTP - Hardware: servers	Technical and semantic	Local	Hospitals	Open MRS
Cassavia N, et all [56]	Between EMR	Web service 1) repository, 2) knowledge discovery module, 3) service modules (collect, aggregate and forward data- data cleaning and data mapping and so on)	Medical data exchange	- Transport: HL7, CORBA and DICOM - Content: CDA and CEN/ISO 13606 - Terminology: SNOMED-CT, RxNorm, ICD 10 and LOINC - Security: OASIS	- Technology: XML, IHE XDS, SSL, SAML and XACML - Software: Apache Hadoop, Flume, HBase, Solr, Lily HBase Indexer and Hue	Syntactic and Semantic	Local	Hospitals	Open EHR
Ebietomere EP et al. [57]	Between EMR	Ontology based 1) extraction of concepts, 2) cleaning of data, 3) ontology creating, 4) evaluation of ontology, 5) data mapping	Medical data exchange	- Transport: HL7 and IEEE 1073 - Terminology: SNOMED-CT, ICD 10, ICD 9 and LOINC	Software: Protégé, excel, Snow Owl, Snoggle, FACT++ OWLViz, ontoGraf and HermiT - Technology: RDF, OWL and SPARQL	Semantic	National	Hospitals	RIM
Dogac A et al. [58]	Between CIS	Web Service 1) national health data dictionary, 2) transmission data sets, 3) mapping the transmission data sets to HL7 CDA schema, 4) health coding reference server, 5) healthcare professional registry, 6) communication infrastructure (cloud), 7) CISs	Data sharing	- Content: ISO/TR 20514:2005, CDA and CEN/ISO 13606 - Transport: HL7 - Terminology: SNOMED-CT, ICD 10, Mesh, READ Codes and LOINC	- Technology: XML and SSL	Structural	Local	Hospitals	RIM

### 3.2 Phase 2. Constructing the framework

In the focus group section, 12 experts took part in this study to finalize the framework. These experts included individuals with expertise in medical informatics (3), health information management/health information technology (3), information technology (2), physicians (2), and nurses (2). Table 2 lists the particulars and attributes of the participants in the focus group sessions.

**Table 2:** Characteristics of the participants in focus groups.

Items		Frequency	Percentage
Gender	Male	11	91.66
	female	1	8.34
Age	20–30	2	16.66
	30–40	5	41.67
	40–50	5	41.67
	>50	0	0
Academic background	Bachelor's degree	2	16.66
	Master's degree	2	16.66
	Ph.D.	8	66.68
Organization	Hospital	6	50
	Company	2	16.66
	University	4	33.34
Job experience	Less than 5 years	0	0
	6–10	2	16.66
	11–15	6	50
	More than 15 years	4	33.34

**Fig. 3:**
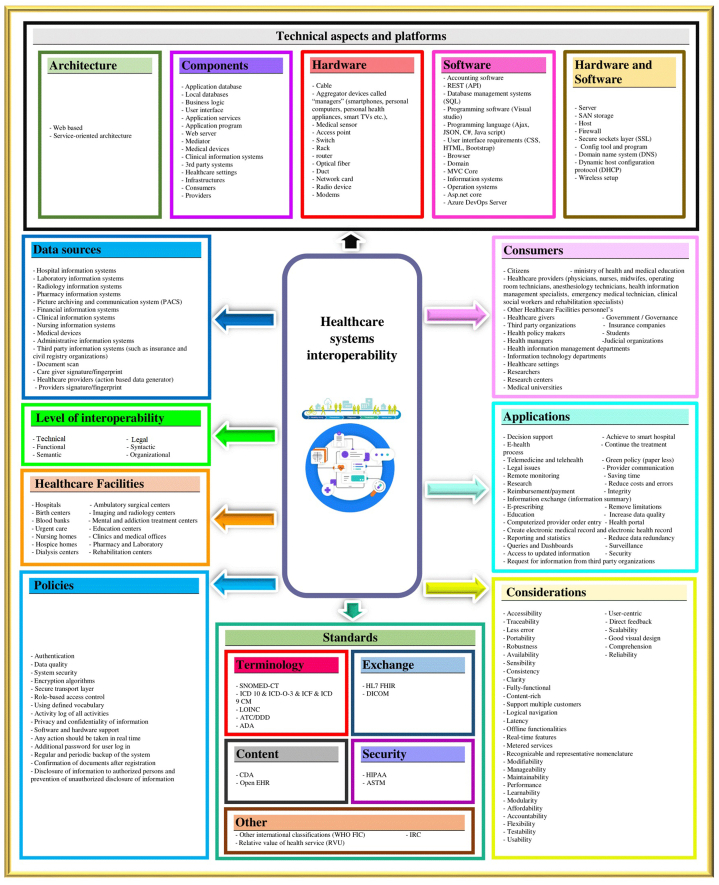
Proposed unified component-based data-driven framework for support interoperability in healthcare systems.

The results of the review and experts' knowledge led to the proposal of a unified, component-based, data-driven framework to support interoperability in healthcare systems, as depicted in Fig. 3. The emerging themes from the findings were categorized into: (1) architecture, (2) architecture components, (3) standards, (4) platforms, (5) Policies, (6) data sources, (7) consumers, (8) applications, (9) level of interoperability, (10) healthcare facilities, and (11) considerations. Additionally, standards were categorized into five sections: terminology, content, exchange, security, and other, and platforms were categorized into three sections: hardware, software, and hardware and software (Refer to Fig. 3 for further details).

**Fig. 4:**
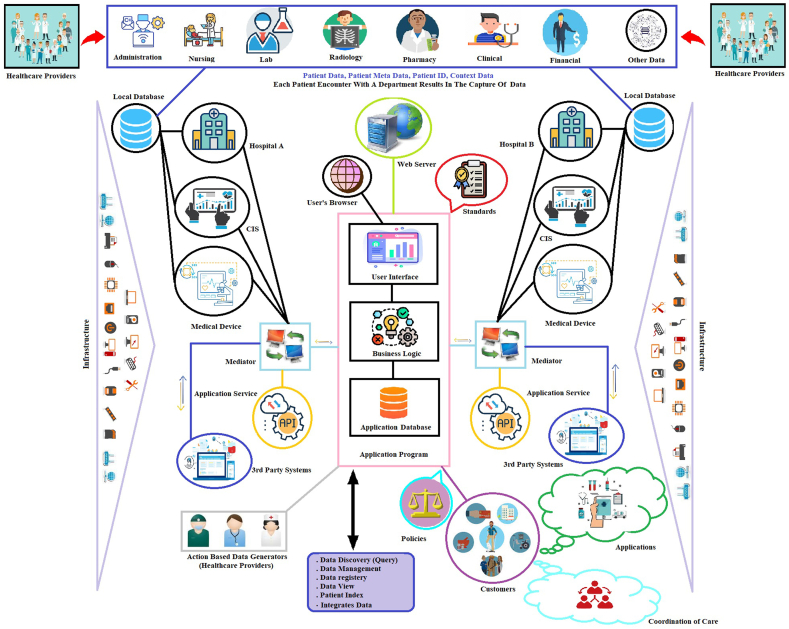
Proposed architecture for support interoperability in healthcare systems.

Clinical experts recommended establishing a comprehensive architecture to facilitate interoperability between health information systems, while technical experts provided a detailed architecture. Fig. 4 illustrates the proposed architecture.

In this architecture, when a patient visits a health service center, a variety of data related to the patient's health (such as laboratory, radiology, pharmacy, administrative, nursing, financial, clinical, etc.) is generated by the health service providers. Additionally, data is also produced from the medical devices connected to the patient and the clinical information systems available in each health service center. These data are stored and managed in local databases within the same center. Any required clinical information that is not available in the aforementioned cases is generated through a web-based application program based on standards by health service providers and stored in a secondary database (web-based application program database). The integration between the information available in the health institutions and the new information produced in the application program is established through middleware and web services. Furthermore, information exchange between third-party organizations (such as insurance, judicial organizations, etc.) with the web-based application program or institutions also follows the same procedure.

In an integrated manner, all available health information can be accessed through the web-based application program in any health institution with queries. Different users can utilize the information for various applications in accordance with established policies. Additionally, third-party organizations can exchange information with health institutions and the application program through web services and middleware, provided that the necessary infrastructure is in place.

### 3.3 Phase 3. Validation of proposed conceptual framework

Out of the 30 experts contacted, 27 responded and completed the survey in the three rounds. Among these specialists, 20 were men and the remaining were women. Additionally, the age distribution was as follows: one person in the 20-to-30-year range, 14 people in the 30-to-40-year range, and 12 people in the 40-to-50-year range. In terms of field of expertise, four respondents were in health information management, two in health information technology, seven in medical informatics, five in information technology, five in medicine, and four in nursing. All of them had more than 10 years of work experience, with 18 working in the university and 9 working in the hospital.

All 197 framework components reflected a 100 % positive consensus. The only concerns raised were about the applicability of certain components, which were resolved with explanations in the third and final round.

## 4 Discussion

In order for multiple applications to operate simultaneously, web-based architecture illustrates the connections between applications, middleware systems, and databases. When a user enters a Uniform Resource Locator (URL) and clicks “Go,” the browser finds the server hosting the website connected to the Internet and requests the specific page [59]. The server then sends files to the browser as a response. Subsequently, the browser processes the files to display the requested page to the user, who can then interact with the website. At this point, the code parsed by the browser becomes significant. This code may or may not contain specific instructions that guide the browser's response to various inputs [60].

A software development approach called service-oriented architecture (SOA) utilizes software components known as services to construct business applications. Each service provides a specific business function and can communicate with other services across different platforms and programming languages. With SOA, developers can integrate multiple distinct services to accomplish complex tasks or reuse services across various systems. Web-based applications are built using SOA at the architectural level [61].

In comparison to traditional monolithic systems, where each process operates as a separate entity, these architectures offer several advantages. They standardize the automation and use of business processes while maintaining security and governance, all while preserving the procedure-call model commonly used in structured programming. Multiple applications can be created using the same services. Organizations can save time and money by reusing services when developing new products [62]. All services can be upgraded and modified without impacting others because they are all independent. This also reduces an organization's operational costs. It enables better adaptation to technological advancements and facilitates the efficient and cost-effective modernization of applications. For example, modern cloud-based applications can leverage features from previous electronic health record systems. Large code chunks are more difficult to debug than smaller services, hence this method results in less dependable programs. It allows services to run on multiple servers, enhancing scalability. Moreover, by employing a standardized communication protocol, enterprises can reduce the amount of interaction between clients and services, enabling applications to be scaled without creating additional stress. Services are available to everyone on request, and platforms can easily convey data between clients and services, regardless of the languages in which they are written. All of these factors ultimately enhance interoperability [63,64].

Ideally, interoperability should naturally develop as a by-product of applying service orientation, to the extent that it is recognized as a common and expected property of service design. The degree of standardization and streamlining of cross-service data flow is frequently closely associated with the accomplishment of composability standards [65].

A service can achieve various levels of interoperability, including foundational, structural, functional, syntactic, semantic, organizational, and others, similar to any other design characteristic. The regular and successful implementation of service-orientation principles, as well as the maturity level of the technology platform, serve as the ultimate yardstick [66].

Considering the advantages and features mentioned and along with the results, Dai and colleagues also used service-oriented architecture in order to achieve interoperability and flexibility of information systems [67].

Most web applications are developed by segmenting their core operations into components or tiers, allowing for quick replacement and upgrade of each component independently. The architecture of a web application encompasses all supporting elements and external application interfaces [68]. Our proposed framework includes application databases, local databases, business logic, user interfaces, application services, application programs, web servers, mediators, clinical information systems, third-party systems, healthcare settings, infrastructures, consumers, and providers as architectural components. Varga et al. also used a component and modular approach to establish interoperability between systems, whose components were similar to the components presented in this study [69].

The hardware and software components must be highly reliable, reconfigurable, and, where necessary, certifiable, from individual components to fully integrated systems. They should be designed using the latest technology to avoid wasteful spending. Recent surveys indicate a 100 % increase in excessive spending on “orphaned” SaaS systems that are no longer in use. In some cases, specialized hardware or software that has not been integrated with other programs or serves only one purpose could go unnoticed for years [70]. Furthermore, the multitude of everyday techniques and remedies sometimes leads to overload. It is not just that the equipment or software is challenging to operate; interoperability can often be made more difficult by incompatible hardware or software. To address these issues, many companies are exploring new approaches to streamline daily hardware or software usage, either by linking unrelated programs or by consolidating various requirements on a single platform [70,71].

Organizations often have a narrow-minded view that creating apps involves only determining an application's capabilities and behavior. Users are not only affected by improper functionality, but also by the absence of essential software quality characteristics that affect the viability of any software solution. A system will inevitably fail if it is not dependable, secure, or scalable, just as if we overlook an important functional need. Quality attributes are one of the two categories of non-functional requirements, commonly regarded as a portion of the job that users cannot see but which benefits them [72]. When it functions properly, it can be considered invisible, but when it does not, users will undoubtedly notice. Several strategies can aid in the pursuit of a specific quality level, but they invariably clash with another attribute. A solid architecture design has tradeoffs. The appropriate attributes for the application, their importance, and how they will affect the other attributes must be determined properly [73].

Various health information systems are used to support daily activities in an environment where they are pervasive. Additionally, businesses can gather operational data in real-time, but these data are continuously delivered to various health information systems. Without processing, data have no value and cannot be used to support decisions. The challenges of data interoperability among challenging data sources are exacerbated by the widespread usage of health information systems and devices inside an organization [74]. Since data sources from apps and devices are characterized by a variety of heterogeneities, including developing settings, communication protocols, and blinding methods, the complexity of handling heterogeneous data sources is further raised. Because information interoperability has a major impact on decision-making efficacy and efficiency, it is crucial in ubiquitous companies [75]. This article introduces a groundbreaking framework for interoperability that serves as middleware to provide a variety of data sources with information integration and querying capabilities.

Why would a user download one version of an app on their computer and another on their phone or other device? The public anticipates interoperability. They want assurance that their programs will follow them and not require additional logins, lose data when switching platforms, or become dysfunctional when using various hardware configurations [76].

Healthcare organizations still lack the regulated setting that is typical of research programs. Existing, historical, and occasionally unstructured data are devoid of any standards or vocabularies. Furthermore, there are duplicate entries in the patient history because there is no integrated system. This situation is made worse at the level of system providers, who maintain proprietary storage structures and prevent data sharing with other institutions without a lengthy period for modifications. Because of this, it is essential to acknowledge reality and present viable and practical solutions [77].

Health standards knowledge is only widely used in academia (educational institutions) and major software suppliers, and it is still not widely used in the market. This means that there is an opportunity to take part by doing things that reduce the recurrent issues with standardization, providing enough materials to support the study, and making information about the resources that are available easily accessible. Since few systems incorporate vocabulary, interchange, content, and security standards into their designs due to the lack of widespread distribution of knowledge about health trends, these elements are not part of the healthcare reality. Thus, it is essential to work on projects that promote the gradual integration of these values into daily life [78].

Interoperability is frequently emphasized by software companies as being essential to integration and information sharing. However, healthcare systems tend to perceive interoperability as a luxury, when it should actually be considered a necessity. This is because interoperability directly impacts the quality of the data that is stored and utilized for future purposes. In order to facilitate data reuse, clinical modeling, conducting research within healthcare institutions, and other essential uses, it is vital to promote the development of systems that prioritize interoperability [79].

In general, various studies have provided a framework for the interoperability of health information systems. For instance, Amin et al. introduced the concept of service-oriented architecture, web service technology, conceptual perspective, logical perspective, physical perspective, as well as modules and entities within the framework they presented [80]. Moreover, Lopez and colleagues outlined generic components, ISO 10746 and HL7 standards, the rational unified process approach (RUP), and service-oriented architecture within their framework [81]. Additionally, Kumar et al. presented a framework in their research that encompasses RIM backbone classes, HL7 standards, a Message Exchanging Model, and XML algorithms [82]. In contrast to the previously mentioned studies, the current research initially conducted an extensive literature review to thoroughly analyze all the challenges and requirements related to interoperability in health information systems. Subsequently, based on the findings, as well as input from a focus group and the Delphi technique, the framework was developed. Additionally, while prior studies have focused on specific aspects like semantic interoperability and message exchange, the present study addresses a wide range of interoperability aspects such as architecture, components, standards, platforms, policies, data sources, consumers, applications, interoperability levels, healthcare facilities, and considerations.

## 5 Implication and limitation

In terms of the beneficiaries, our framework offers significant potential value to multiple stakeholders within the healthcare ecosystem. First and foremost, healthcare providers, including hospitals, clinics, and medical practitioners, can benefit from improved interoperability by seamlessly exchanging patient data across different systems. This enhanced information sharing can lead to ease and increase access to integrated, timely and complete information about patients, improve communication between providers, more efficient and informed decision-making, and better patient care. Also, due to saving time and money, and increasing patient participation in self-care, patient satisfaction increases [79]. Additionally, healthcare IT professionals and system administrators can leverage our framework to design and implement interoperable systems, leading to streamlined processes, decreased administrative burdens, and enhanced system efficiency. This empowers them to efficiently manage healthcare data and improve overall system performance. Policymakers and healthcare regulators are also beneficiaries of this study. By understanding the advantages of our framework, they can make informed decisions to develop and enforce robust interoperability regulations and standards, facilitate medical education, facilitate health research, improve health care oversight, reduce time and financial costs, and foster a more connected and patient-centric healthcare landscape [83].

We acknowledge that the healthcare sector encompasses numerous complexities, such as diverse data formats, privacy concerns, regulatory requirements, and varying levels of IT infrastructure across different healthcare organizations. We acknowledge that these difficulties are intrinsic elements that could influence how well our framework is adopted and put into practice. By outlining and discussing these challenges, we ensure a comprehensive understanding of the practical considerations associated with its implementation in various healthcare settings. Recognizing the scope and resources of our research, we openly acknowledge certain constraints that may impact the generalization and applicability of our findings. For instance, due to time and resource constraints, implementation and evaluation of our framework has been conducted in a limited number of healthcare institutions, potentially limiting the generalizability of our results. It is important to note that these limitations serve as opportunities for future research and improvements within the domain of healthcare interoperability.

The recommendations for establishing a roadmap to interoperability include:

• Assessing the current state of information systems in medical institutions.
• Implementing architectures and components that do not require modifications to existing systems or impose significant additional burdens on them, such as service-oriented architecture and web-based solutions.
• Utilizing adaptable and scalable development platforms to accommodate future changes and advancements, aligning with the platforms suggested in this study.
• Taking into account all levels of interoperability, applications, stakeholders, and data sources.
• Addressing both functional and non-functional policies and requirements, as outlined in the proposed framework.
• Emphasizing the healthcare informatics market's focus on adhering to global standards, including those recommended in this study.
• Giving particular consideration to security issues, information confidentiality, and personal privacy.

## 6 Conclusion

This study aimed to develop a unified, component-based, data-driven framework to solve interoperability challenges in healthcare systems. Patient-centered and data-driven care delivery is replacing disease-centered care delivery in the healthcare industry. Personalized healthcare systems are also in greater demand. This calls for more interoperability among all healthcare actors, especially when all the varying types of data need to interact. The literature review made it possible to examine the existing approaches taken to achieve interoperability at all levels. Furthermore, we stress the importance of interoperability at the healthcare organization level, considering the difficulties in integrating, sharing, and transferring health records among all business units. Interoperability requires organizational participation from all levels of the participating teams, including IT staff and medical professionals, as well as from institutional administration. Consequently, it is critical to recognize the reality of healthcare facilities, where patients are respected for their current state of health and are reflected in all interactions and processes through the delivery of high-quality, cost-effective care. We think that our framework can help healthcare systems implement various forms of interoperability more easily. By ensuring its seamless integration into existing healthcare systems, due to the extensive capabilities of interoperability, firstly, we emphasize the importance of conducting empirical studies and evaluations involving various healthcare institutions. By engaging in collaborative research efforts with different stakeholders, such as healthcare providers, administrators, and IT professionals, we aim to assess the framework's performance, scalability, and compatibility in a variety of real-world healthcare environments. This approach enables us to gather diverse perspectives and address potential limitations or challenges that may arise in practical implementations. Additionally, we highlight the significance of conducting usability studies to assess the framework's user experience and adoption. By incorporating user feedback and considering the unique needs and preferences of healthcare professionals, we can refine the framework's design and functionality, ensuring its seamless integration into existing healthcare systems. Lastly, we acknowledge the need for continuous updates and enhancements to ensure the framework remains adaptable to shifting technological landscapes, evolving healthcare standards, and emerging data management requirements. We address the importance of actively engaging with the healthcare community, standards organizations, and policymakers to stay current with industry trends and incorporate relevant advancements into our framework design.

## Ethics approval and consent to participate

All the methods were performed in accordance with relevant guidelines and regulations. Before the study was conducted, all participants received an information statement about the study and provided written consent to participate. This study was approved by the Ethics Committee of Tabriz University of Medical Sciences (IR.TBZMED.REC.1401.488).

## Funding

Not applicable.

## Data availability statement

Data will be made available on request.

## CRediT authorship contribution statement

**Amir Torab-Miandoab:** Writing – review & editing, Writing – original draft, Visualization, Validation, Software, Resources, Methodology, Investigation, Formal analysis, Data curation, Conceptualization. **Taha Samad-Soltani:** Writing – review & editing, Writing – original draft, Validation, Software, Methodology, Investigation, Formal analysis, Data curation. **Ahmadreza Jodati:** Validation, Resources, Methodology, Investigation, Data curation, Conceptualization. **Fariborz Akbarzadeh:** Validation, Resources, Methodology, Formal analysis, Data curation. **Peyman Rezaei-Hachesu:** Writing – review & editing, Visualization, Validation, Supervision, Software, Project administration, Methodology, Investigation, Formal analysis, Data curation, Conceptualization.

## Declaration of competing interest

The authors declare that they have no known competing financial interests or personal relationships that could have appeared to influence the work reported in this paper.

## List of Abbreviations

ICT: Information and Communications Technology
EHR: Electronic Health Record
EMR: Electronic Medical Record
PHR: Patient Health Record
SNOMED-CT: Systematized Nomenclature of Medicine-Clinical Terms
ICD: International Classification of Diseases
CPT: Current Procedural Terminology
ABC: Alternative Billing Codes
NANDA: North American Nursing Diagnosis Association
NOC: Nursing Outcomes Classification
NIC: Nursing Interventions Classification
LOINC: Logical Observation Identifiers Names and Codes
HCPCS: Healthcare Common Procedure Coding System
CCC: Clinical Care classification
CDA: clinical document architecture
HL7: Health Level Seven
HIPAA: Health Insurance Portability and Accountability Act
PC: Personal Computer
XML: Extensible Markup Language
SOA: Service-Oriented Architecture
SOAP: Simple Object Access Protocol
UDDI: Universal Description Discovery and Integration
WSDL: Web services Definition Language
PDA: Personal Digital Assistant
ADSL: Asymmetric digital subscriber line
RMI: Remote Method Invocation
NHIN: Nationwide Health Information Network
SANDS: Service-Oriented Architecture for NHIN Decision Support
REST: Representational state transfer
RFID: Radio Frequency Identification devices
PAN: personal area network
CRM: Conceptual Reference Model
ATC: Anatomical Therapeutic Chemical
AIC: Akaike Information Criterion
FHIR: Fast Healthcare Interoperability Resources
SOAP: Simple Object Access Protocol
IHE: Integrating the Healthcare Enterprise
ITI: Information Technology Infrastructure
IHE XDS: Integrating the Healthcare Enterprise Extended Data Services
RM: Reference model
ADL: Archetype Definition Language
SAML: Security Assertion Markup Language
AOM: Archetype Object Model
ISO: International Organization for Standardization
LDAP: Lightweight Directory Access Protocol
DICOM: Digital Imaging and Communications in Medicine
NDC: National Drug Code
GDPR: General Data Protection Regulation
TCP: Transmission Control Protocol
API: Application Programming Interface
IOT: Internet of Things
HIS: Hospital (Health) Information Systems
WSDM: Web Service of Data Mediation
UMLS: Unified Medical Language System
EDIFACT: Electronic Data Interchange for Administration, Commerce and Transport
RIM: Reference Information Model
CDR: Clinical Documents Repository
ICPC: International Classification of Primary Care
AQL: ArangoDB Query Language
SQL: Structured Query Language
JSON: JavaScript Object Notation
CIS: Clinical Information System
CCR: Continuity of Care Record
PDF: Portable Document Format
SVG: Scalable Vector Graphics
URL: Uniform Resource Locator
CCD: Continuity of Care Document
LIS: Laboratory Information Systems
CORBA: The Common Object Request Broker Architecture
OMG: Object Management Group's
COM: Component Object Model
OWL: Web Ontology Language
RDF: Resource Description Framework
DSS: Decision Support System
OQL: Object Query Language
HTML: Hyper Text Markup Language
NCPDP: National Council on Prescription Drug Programs
IETF: Internet Engineering Task Force
ASTM: American Society for Testing and materials
Open MRS: Open Medical Record System
HTTP: Hypertext Transfer Protocol
OAuth: Open Standard for Authorization
PACS: Picture Archiving and Communication Systems
CDS: Clinical Decision Support
Continua: Continua Health Alliance
UML: Unified Modeling Language
ANSI: American National Standards Institute
HITECH: Health Information Technology for Economic and Clinical Health
CORBA: Common Object Request Broker Architecture
OASIS: Organization for the Advancement of Structured Information Standards
SSL: Secure Socket Layer
HTTPS: Hypertext Transfer Protocol Over Secure Socket Layer
XACML: Extensible Access Control Markup Language
SAML: Security Assertion Markup Language
RUP: Rational Unified Process
