# Supplementary material for: A unified component-based data-driven framework to support interoperability in the healthcare systems
Source: Heliyon. 2024 Jul 23;10(15):e35036. doi: 10.1016/j.heliyon.2024.e35036 (PMC11332873; doi:10.1016/j.heliyon.2024.e35036)
Supplement: Multimedia component 2 [file mmc2.pdf]

Quality Assessment of Diagnostic Accuracy Studies (QUADAS)

Appendix E lists the questions asked to conduct the quality assessment of the included studies using the QUADAS tool.

Total QUADAS score range = **0 – 14**

1. Was the spectrum of patients representative of the patients who will receive the test in practice?

- Yes (score = 1)
- No (score = 0)
- Unclear (score = 0)

2. Were selection criteria clearly described?

- Yes (score = 1)
- No (score = 0)
- Unclear (score = 0)

3. Is the reference standard and index test short enough to be reasonably sure that the target condition did not change between the two tests?

- Yes (score = 1)
- No (score = 0)
- Unclear (score = 0)

4. Is the time period between reference standard and index test short enough to be reasonably sure that the target condition did not change between the two tests?

- Yes (score = 1)
- No (score = 0)
- Unclear (score = 0)

5. Did the whole sample or a random selection of the sample, receive verification using a reference standard of diagnosis?

- Yes (score = 1)
- No (score = 0)
- Unclear (score = 0)

6. Did patients receive the same reference standard regardless of the index test result?

- Yes (score = 1)
- No (score = 0)
- Unclear (score = 0)

7. Was the reference standard independent of the index test (i.e. the index test did not form part of the reference standard)?

- Yes (score = 1)
- No (score = 0)
- Unclear (score = 0)

8. Was the execution of the index test described in sufficient detail to permit replication of the test?

- Yes (score = 1)
- No (score = 0)
- Unclear (score = 0)

9. Was the execution of the reference standard described in sufficient detail to permit its replication?

- Yes (score = 1)
- No (score = 0)
- Unclear (score = 0)

10. Were the index test results interpreted without knowledge of the results of the reference standard?

- Yes (score = 1)
- No (score = 0)
- Unclear (score = 0)

11. Were the reference standard results interpreted without knowledge of the results of the index test?

- Yes (score = 1)
- No (score = 0)

- Unclear (score = 0)

12. Were the same clinical data available when test results were interpreted as would be available when the test is used in practice?

- Yes (score = 1)
- No (score = 0)
- Unclear (score = 0)

13. Were uninterpretable/ intermediate test results reported?

- Yes (score = 1)
- No (score = 0)
- Unclear (score = 0)

14. Were withdrawals from the study explained?

- Yes (score = 1)
- No (score = 0)
- Unclear (score = 0)
