# Supplementary material for: A unified component-based data-driven framework to support interoperability in the healthcare systems
Source: Heliyon. 2024 Jul 23;10(15):e35036. doi: 10.1016/j.heliyon.2024.e35036 (PMC11332873; doi:10.1016/j.heliyon.2024.e35036)
Supplement: Multimedia component 3 [file mmc3.pdf]

Quality Appraisal of Diagnostic Reliability (QAREL) Checklist

Item		Yes	No	Unclear	N/A
1.	Was the test evaluated in a sample of subjects who were representative of those to whom the authors intended the results to be applied?<br>(DEF: 3, 4, 5, 7, 8, 9)	☐	☐	☐	
2.	Was the test performed by raters who were representative of those to whom the authors intended the results to be applied?<br>(DEF 3, 4, 6, 7, 8, 9)	☐	☐	☐	
3.	Were raters blinded to the findings of other raters during the study?<br>(DEF 10)	☐	☐	☐	☐
4.	Were raters blinded to their own prior findings of the test under evaluation?<br>(DEF 11)	☐	☐	☐	☐
5.	Were raters blinded to the results of the reference standard for the target disorder (or variable) being evaluated?<br>(DEF 12)	☐	☐	☐	☐
6.	Were raters blinded to clinical information that was not intended to be provided as part of the testing procedure or study design?<br>(DEF 13)	☐	☐	☐	☐
7.	Were raters blinded to additional cues that were not part of the test?<br>(DEF 14)	☐	☐	☐	
8.	Was the order of examination varied?<br>(DEF 15, 16)	☐	☐	☐	☐
9.	Was the time interval between repeated measurements compatible with the stability (or theoretical stability) of the variable being measured?<br>(DEF 17)	☐	☐	☐	
10.	Was the test applied correctly and interpreted appropriately?<br>(DEF 18)	☐	☐	☐	
11.	Were appropriate statistical measures of agreement used?<br>(DEF 19, 20, 21)	☐	☐	☐	
	**TOTAL**				

DEF numbers relate to items on the QAREL Data Extraction Form
To access the Data Extraction Form, please go to http://qarel.org

The Quality Appraisal of Reliability Checklist
© 2012 Nicholas Lucas | All Rights Reserved | QAREL.ORG

Please cite all usage of this checklist as, Lucas NP, Macaskill P, Irwig I, Bogduk N. The development of a quality appraisal tool for studies of diagnostic reliability. *Journal of Clinical Epidemiology*, 2010; 63: 854-861.
