# Supplementary material for: A unified component-based data-driven framework to support interoperability in the healthcare systems
Source: Heliyon. 2024 Jul 23;10(15):e35036. doi: 10.1016/j.heliyon.2024.e35036 (PMC11332873; doi:10.1016/j.heliyon.2024.e35036)
Supplement: Multimedia component 4 [file mmc4.docx]

This study aims to develop an integrated, component-based and data-driven framework for solving interoperability challenges in healthcare systems. Dear experts, please express your opinion about each of the following topics:

(1) Architectures to be used.

(2) Components that the architectures used must have.

(3) Standards to be used.

(4) Platforms that are needed for development and implementation.

(5) Policies to be used.

(6) Data sources to be considered.

(7) Consumers to be considered.

(8) Applications to be considered.

(9) Level of interoperability to be considered.

(10) Health care facilities that should be considered.

(11) Considerations to be taken into account.

(12) Functional and non-functional requirements to be considered.

In addition to the above, state anything that you think is useful for the purpose of the study.

Thank you
