# Supplementary material for: A unified component-based data-driven framework to support interoperability in the healthcare systems
Source: Heliyon. 2024 Jul 23;10(15):e35036. doi: 10.1016/j.heliyon.2024.e35036 (PMC11332873; doi:10.1016/j.heliyon.2024.e35036)
Supplement: Multimedia component 5 [file mmc5.docx]

|  | All Elements | Totally relevant | Slightly related | No idea | Slightly unrelated | Totally unrelated |
| --- | --- | --- | --- | --- | --- | --- |
| Architecture | - Web based |  |  |  |  |  |
|  | - Service-oriented architecture |  |  |  |  |  |
| Components | - Application database |  |  |  |  |  |
|  | - Local databases |  |  |  |  |  |
|  | - Business logic |  |  |  |  |  |
|  | - User interface |  |  |  |  |  |
|  | - Application services |  |  |  |  |  |
|  | - Application program |  |  |  |  |  |
|  | - Web server |  |  |  |  |  |
|  | - Mediator |  |  |  |  |  |
|  | - Medical devices |  |  |  |  |  |
|  | - Clinical information systems |  |  |  |  |  |
|  | - 3rd party systems |  |  |  |  |  |
|  | - Healthcare settings |  |  |  |  |  |
|  | - Infrastructures |  |  |  |  |  |
|  | - Consumers |  |  |  |  |  |
|  | - Providers |  |  |  |  |  |
| Technical aspects and platforms | - Cable |  |  |  |  |  |
|  | - Aggregator devices called “managers” (smartphones, personal computers, personal health appliances, smart TVs etc.), |  |  |  |  |  |
|  | - Medical sensor |  |  |  |  |  |
|  | - Access point |  |  |  |  |  |
|  | - Switch |  |  |  |  |  |
|  | - Rack |  |  |  |  |  |
|  | - router |  |  |  |  |  |
|  | - Optical fiber |  |  |  |  |  |
|  | - Duct |  |  |  |  |  |
|  | - Network card |  |  |  |  |  |
|  | - Radio device |  |  |  |  |  |
|  | - Modems |  |  |  |  |  |
|  | - Accounting software |  |  |  |  |  |
|  | - REST (API) |  |  |  |  |  |
|  | - Database management systems (SQL) |  |  |  |  |  |
|  | - Programming software (Visual studio) |  |  |  |  |  |
|  | - Programming language (Ajax, JSON, C#, Java script) - User interface requirements (CSS, HTML, Bootstrap) |  |  |  |  |  |
|  | - Browser |  |  |  |  |  |
|  | - Domain |  |  |  |  |  |
|  | - MVC Core |  |  |  |  |  |
|  | - Information systems |  |  |  |  |  |
|  | - Operation systems |  |  |  |  |  |
|  | - Asp.net core |  |  |  |  |  |
|  | - Azure DevOps Server |  |  |  |  |  |
|  | - Accounting software |  |  |  |  |  |
|  | - Server |  |  |  |  |  |
|  | - SAN storage |  |  |  |  |  |
|  | - Host |  |  |  |  |  |
|  | - Firewall |  |  |  |  |  |
|  | - Secure sockets layer (SSL) |  |  |  |  |  |
|  | - Config tool and program |  |  |  |  |  |
|  | - Domain name system (DNS) |  |  |  |  |  |
|  | - Dynamic host configuration protocol (DHCP) |  |  |  |  |  |
|  | - Wireless setup |  |  |  |  |  |
| Data sources | - Hospital information systems |  |  |  |  |  |
|  | - Laboratory information systems |  |  |  |  |  |
|  | - Radiology information systems |  |  |  |  |  |
|  | - Pharmacy information systems |  |  |  |  |  |
|  | - Picture archiving and communication system (PACS) |  |  |  |  |  |
|  | - Financial information systems |  |  |  |  |  |
|  | - Clinical information systems |  |  |  |  |  |
|  | - Nursing information systems |  |  |  |  |  |
|  | - Medical devices |  |  |  |  |  |
|  | - Administrative information systems |  |  |  |  |  |
|  | - Third party information systems (such as insurance and civil registry organizations) |  |  |  |  |  |
|  | - Document scan |  |  |  |  |  |
|  | - Care giver signature/fingerprint |  |  |  |  |  |
|  | - Healthcare providers (action based data generator) |  |  |  |  |  |
|  | - Providers signature/fingerprint |  |  |  |  |  |
| interoperability | - Foundational |  |  |  |  |  |
|  | - Functional |  |  |  |  |  |
|  | - Semantic |  |  |  |  |  |
|  | - Structural |  |  |  |  |  |
|  | - Syntactic |  |  |  |  |  |
|  | - Organizational |  |  |  |  |  |
| Facilities | - Hospitals |  |  |  |  |  |
|  | - Ambulatory surgical centers |  |  |  |  |  |
|  | - Birth centers |  |  |  |  |  |
|  | - Imaging and radiology centers |  |  |  |  |  |
|  | - Blood banks |  |  |  |  |  |
|  | - Mental and addiction treatment centers |  |  |  |  |  |
|  | - Urgent care |  |  |  |  |  |
|  | - Education centers |  |  |  |  |  |
|  | - Nursing homes |  |  |  |  |  |
|  | - Clinics and medical offices |  |  |  |  |  |
|  | - Hospice homes |  |  |  |  |  |
|  | - Pharmacy and Laboratory |  |  |  |  |  |
|  | - Dialysis centers |  |  |  |  |  |
|  | - Rehabilitation centers |  |  |  |  |  |
| Policies | - Authentication |  |  |  |  |  |
|  | - Data quality |  |  |  |  |  |
|  | - System security |  |  |  |  |  |
|  | - Encryption algorithms |  |  |  |  |  |
|  | - Secure transport layer |  |  |  |  |  |
|  | - Role-based access control |  |  |  |  |  |
|  | - Using defined vocabulary |  |  |  |  |  |
|  | - Activity log of all activities |  |  |  |  |  |
|  | - Privacy and confidentiality of information |  |  |  |  |  |
|  | - Software and hardware support |  |  |  |  |  |
|  | - Any action should be taken in real time |  |  |  |  |  |
|  | - Additional password for user log in |  |  |  |  |  |
|  | - Regular and periodic backup of the system |  |  |  |  |  |
|  | - Confirmation of documents after registration |  |  |  |  |  |
|  | - Disclosure of information to authorized persons and prevention of unauthorized disclosure of information |  |  |  |  |  |
| Standards | - SNOMED-CT |  |  |  |  |  |
|  | - ICD 10 & ICD-O-3 & ICF & ICD 9 CM |  |  |  |  |  |
|  | - LOINC |  |  |  |  |  |
|  | - ATC/DDD |  |  |  |  |  |
|  | - ADA |  |  |  |  |  |
|  | - HL7 FHIR |  |  |  |  |  |
|  | - DICOM |  |  |  |  |  |
|  | - CDA |  |  |  |  |  |
|  | - Open EHR |  |  |  |  |  |
|  | - HIPAA |  |  |  |  |  |
|  | - ASTM |  |  |  |  |  |
|  | - Other international classifications (WHO FIC) |  |  |  |  |  |
|  | - IRC |  |  |  |  |  |
|  | - Relative value of health service (RVU) |  |  |  |  |  |
| Consumers | - Citizens |  |  |  |  |  |
|  | - ministry of health and medical education |  |  |  |  |  |
|  | - Healthcare providers (physicians, nurses, midwifes, operating room technicians, anesthesiology technicians, health information management specialists, emergency medical technician, clinical social workers and rehabilitation specialists) |  |  |  |  |  |
|  | - Other Healthcare Facilities personnel’s |  |  |  |  |  |
|  | - Healthcare givers |  |  |  |  |  |
|  | - Government / Governance |  |  |  |  |  |
|  | - Third party organizations |  |  |  |  |  |
|  | - Insurance companies |  |  |  |  |  |
|  | - Health policy makers |  |  |  |  |  |
|  | - Students |  |  |  |  |  |
|  | - Health managers |  |  |  |  |  |
|  | - Judicial organizations |  |  |  |  |  |
|  | - Health information management departments |  |  |  |  |  |
|  | - Information technology departments |  |  |  |  |  |
|  | - Healthcare settings |  |  |  |  |  |
|  | - Researchers |  |  |  |  |  |
|  | - Research centers |  |  |  |  |  |
|  | - Medical universities |  |  |  |  |  |
| Applications | - Decision support |  |  |  |  |  |
|  | - Achieve to smart hospital |  |  |  |  |  |
|  | - E-health |  |  |  |  |  |
|  | - Continue the treatment process |  |  |  |  |  |
|  | - Telemedicine and telehealth |  |  |  |  |  |
|  | - Green policy (paper less) |  |  |  |  |  |
|  | - Legal issues |  |  |  |  |  |
|  | - Provider communication |  |  |  |  |  |
|  | - Remote monitoring |  |  |  |  |  |
|  | - Saving time |  |  |  |  |  |
|  | - Research |  |  |  |  |  |
|  | - Reduce costs and errors |  |  |  |  |  |
|  | - Reimbursement/payment |  |  |  |  |  |
|  | - Integrity |  |  |  |  |  |
|  | - Information exchange (information summary) |  |  |  |  |  |
|  | - E-prescribing |  |  |  |  |  |
|  | - Remove limitations |  |  |  |  |  |
|  | - Education |  |  |  |  |  |
|  | - Increase data quality |  |  |  |  |  |
|  | - Computerized provider order entry |  |  |  |  |  |
|  | - Health portal |  |  |  |  |  |
|  | - Create electronic medical record and electronic health record |  |  |  |  |  |
|  | - Reporting and statistics |  |  |  |  |  |
|  | - Reduce data redundancy |  |  |  |  |  |
|  | - Queries and Dashboards |  |  |  |  |  |
|  | - Surveillance |  |  |  |  |  |
|  | - Access to updated information |  |  |  |  |  |
|  | - Security |  |  |  |  |  |
|  | - Request for information from third party organizations |  |  |  |  |  |
| Considerations (non – functional) | - Accessibility |  |  |  |  |  |
|  | - User-centric |  |  |  |  |  |
|  | - Traceability |  |  |  |  |  |
|  | - Direct feedback |  |  |  |  |  |
|  | - Less error |  |  |  |  |  |
|  | - Scalability |  |  |  |  |  |
|  | - Portability |  |  |  |  |  |
|  | - Good visual design |  |  |  |  |  |
|  | - Robustness |  |  |  |  |  |
|  | - Comprehension |  |  |  |  |  |
|  | - Availability |  |  |  |  |  |
|  | - Reliability |  |  |  |  |  |
|  | - Sensibility |  |  |  |  |  |
|  | - Consistency |  |  |  |  |  |
|  | - Clarity |  |  |  |  |  |
|  | - Fully-functional |  |  |  |  |  |
|  | - Content-rich |  |  |  |  |  |
|  | - Support multiple customers |  |  |  |  |  |
|  | - Logical navigation |  |  |  |  |  |
|  | - Latency |  |  |  |  |  |
|  | - Offline functionalities |  |  |  |  |  |
|  | - Real-time features |  |  |  |  |  |
|  | - Metered services |  |  |  |  |  |
|  | - Recognizable and representative nomenclature |  |  |  |  |  |
|  | - Modifiability |  |  |  |  |  |
|  | - Manageability |  |  |  |  |  |
|  | - Maintainability |  |  |  |  |  |
|  | - Performance |  |  |  |  |  |
|  | - Learnability |  |  |  |  |  |
|  | - Modularity |  |  |  |  |  |
|  | - Affordability |  |  |  |  |  |
|  | - Accountability |  |  |  |  |  |
|  | - Flexibility |  |  |  |  |  |
|  | - Testability |  |  |  |  |  |
|  | - Usability |  |  |  |  |  |

In addition to the above, state anything that you think is useful for the purpose of the study.
